# Transcatheter heart valve commissural alignment: an updated review

**DOI:** 10.3389/fcvm.2023.1154556

**Published:** 2023-04-19

**Authors:** Mariama Akodad, Youcef Lounes, David Meier, Francesca Sanguineti, Thomas Hovasse, Philipp Blanke, Janarthanan Sathananthan, Georgios Tzimas, Jonathon Leipsic, David A. Wood, John Webb, Bernard Chevalier

**Affiliations:** ^1^ Ramsay Générale de Santé, Institut Cardiovasculaire Paris Sud, Interventional Cardiology Department, Massy, France; ^2^ Ramsay Générale de Santé, Institut Cardiovasculaire Paris Sud, Vascular Surgery Department, Massy, France; ^3^ Division of Cardiology and Department of Radiology, Centres for Heart Valve Innovation and for Cardiovascular Innovation, St Paul’s and Vancouver General Hospitals, University of British Columbia, Vancouver, BC, Canada

**Keywords:** THV, TAVR, coronary access, redo TAVR, commissural alignment

## Abstract

Transcatheter aortic valve replacement (TAVR) indications recently extended to lower surgical risk patients with longer life expectancy. Commissural alignment (CA) is one of the emerging concepts and is becoming one of the cornerstones of the TAVR procedure in a patient with increased longevity. Indeed, CA may improve transcatheter heart valve (THV) hemodynamics, future coronary access, and repeatability. The definition of CA has been recently standardized by the ALIGN-TAVR consortium using a four-tier scale based on CT analysis. Progress has been made during the index TAVR procedure to optimize CA, especially with self-expandable platforms. Indeed, specific delivery catheter orientation, THV rotation, and computed-tomography-derived views have been proposed to achieve a reasonable degree of CA. Recent data demonstrate feasibility, safety, and a significant reduction in coronary overlap using these techniques, especially with self-expandable platforms. This review provides an overview of THV CA including assessment methods, alignment techniques during the index TAVR procedure with different THV platforms, the clinical impact of commissural misalignment, and challenging situations for CA.

## Introduction

Transcatheter aortic valve replacement (TAVR) is a well-established therapeutic option for severe aortic stenosis. According to current guidelines, TAVR indications can be extended to lower-surgical-risk patients with longer life expectancy (1). Thus, new concepts for TAVR procedure improvement have emerged as they may impact long-term outcomes in younger TAVR populations (2). Optimal transcatheter heart valve (THV) hemodynamics, future coronary access, and repeatability have been of primary importance (3, 4). Similarly to surgical bioprosthetic valves, THVs may eventually degenerate, and repeat intervention may be required. In some patients, repeat procedures might be associated with a high risk of coronary obstruction due to anatomical and/or device considerations. Commissural alignment (CA), routinely achieved with surgical aortic valve replacement, may improve future coronary access (5, 6). Moreover, CA may improve THV hemodynamics and reduce the risk of hypoattenuated leaflet thickening (HALT) (5, 6).

Specific THV and delivery catheter orientations have been proposed during the index TAVR procedure to improve CA. Recent research demonstrates that preprocedural planning is key to achieving CA, demonstrating feasibility, safety, and advantages in reducing coronary overlap. CA feasibility and benefits are currently better documented for self-expandable devices but may be similar to balloon-expandable THVs (6–9).

This review provides an overview of THV CA including assessment methods, alignment techniques during the index TAVR procedure with different THV platforms, clinical impact of commissural misalignment, and challenging situations for CA.

## Commissural alignment with current THV platforms

### Self-expandable THVs

#### Evolut platform

**Figure 1:**
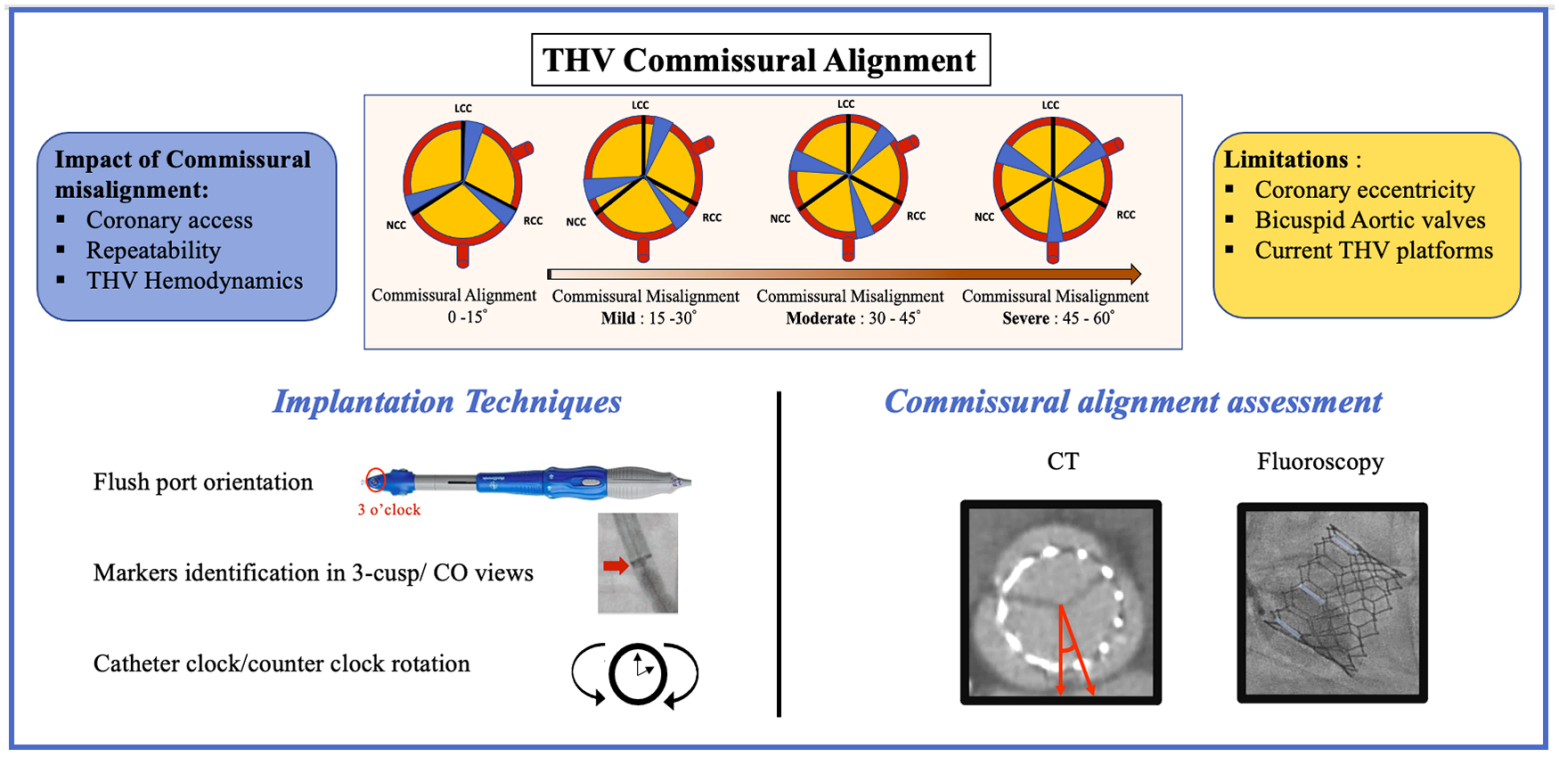
THV commissural alignment: classification, clinical impact, limitations, implantation techniques, and commissural alignment assessment with imaging. CT, computed tomography; CO, cusp overlap; THV, transcatheter heart valve.

**Figure 2:**
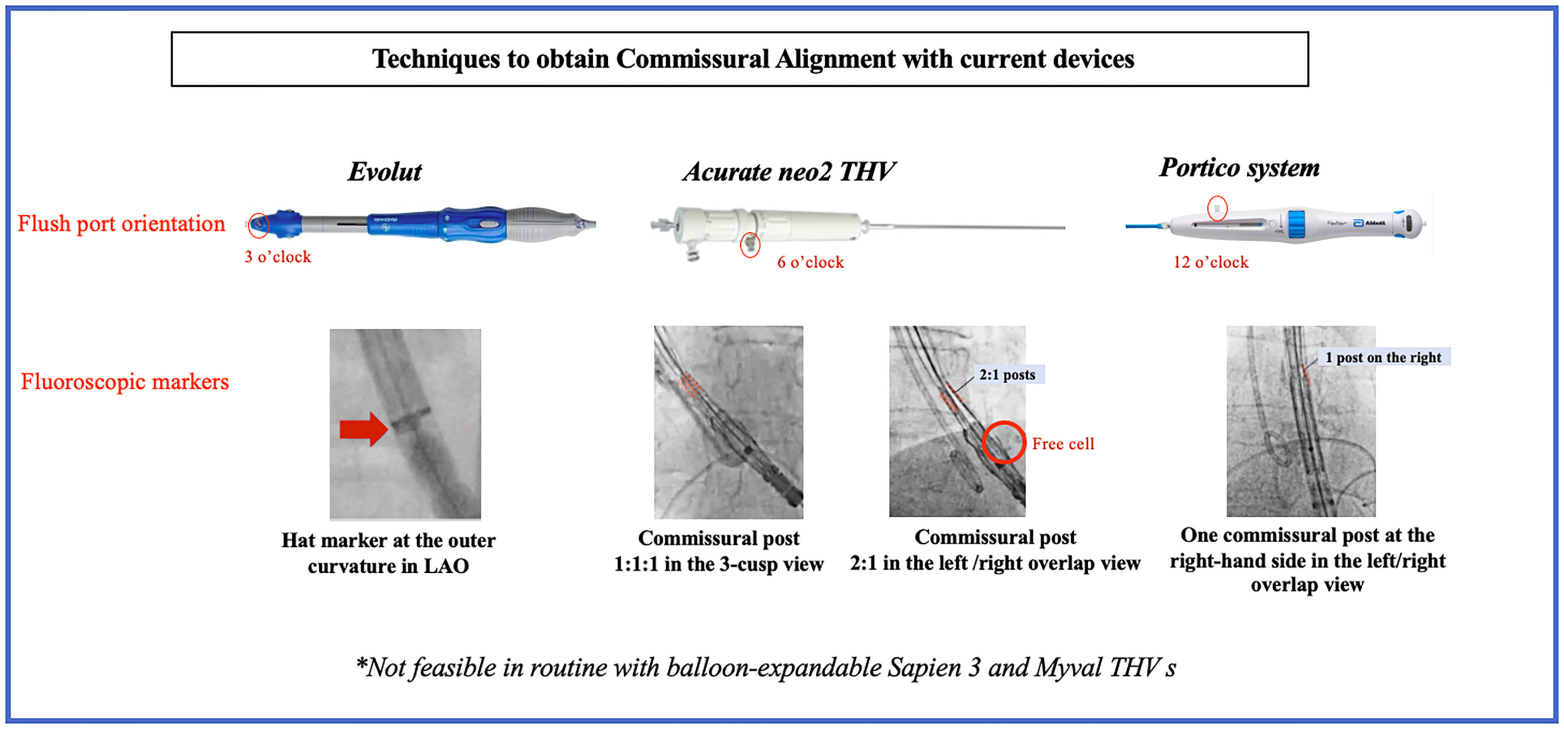
Techniques to obtain commissural alignment with current devices. LAO, left anterior oblique; THV, transcatheter heart valve.

The commissural alignment technique during the index TAVR procedure has been first described with the Evolut (Medtronic, Minneapolis, Minnesota) platform by inserting the delivery catheter with the flush port positioned at 3 O’clock and then orientating the hat marker (90° counterclockwise away from the C-paddle corresponding to one of the THV commissures) in the outer curvature of the descending aorta before final position (Figures 1, 2). These maneuvers aim to orientate the hat marker at the outer curvature or the center front in the 3-cusp coplanar view (right coronary cusp-centered view) and the center front in the right and left coronary cusp overlap view (Figures 1, 2) (10). This technique could be performed in 85.9% of patients (*n* = 64) in the ALIGN-ACCESS study (7).

#### Acurate *neo2* THV

The delivery catheter is inserted with the flush port at 6 O’clock with the Acurate *neo2* (Acn2) valve (Boston Scientific Corporation, Natick, MA, USA). The THV commissures can be identified using the posts on the stabilizing arches and/or the free cells located on the lower crown. The valve is then checked in the 3-cusp view and rotated 5–10 mm above the annulus plane to achieve the desired position. The Acn2 commissural post configuration should be 1-1-1 in the 3-cusp view (clockwise or counterclockwise maneuvers allow to achieve a 1-1-1 configuration if the initial position is 2–1 or 1–2) and 2-1 in the cusp-overlap view (Figure 2) (11, 12). Valve alignment was achieved successfully in 88.5% of patients (*n* = 26) in the ALIGN-ACCESS study (7).

#### Portico system

To optimize CA with the Portico (Abbott Structural Heart) THV, the flush port of the delivery system was inserted at 12 O'clock. The commissural posts can be identified halfway between the THV inflow and outflow. Both the 3-cusp and the right/left cusp-overlap views were used to identify and position one commissural post at the right-hand side in the cusp-overlap view allowing for CA (Figure 2) (11).

### Balloon-expandable THVs

#### Sapien 3 THV

As opposed to self-expandable THVs for which specific techniques have been proposed to achieve CA, CA with the balloon-expandable SAPIEN 3 (S3) (Edwards Lifesciences, Irvine, CA, USA) THV is not currently feasible. Only one study assessed different crimping positions with this THV and failed to demonstrate any crimping position allowing to achieve CA (7).

#### Myval THV

One study has recently shown the feasibility of CA using the Myval balloon-expandable THV in 10 patients using an in silico simulation with correct alignment in all patients with the *in vivo* procedures demonstrating correct alignment in six of the 10 patients and mild misalignment in the remaining four patients. In this study, the authors simulated the orientation of the THV in an in silico model and then adapted the THV crimping to achieve the predicted angle for CA using pre-TAVR computed tomography (CT) for a tailored approach (13).

Although these techniques have been described to optimize CA, some limitations should be considered. First, iliofemoral tortuosity, aorta tortuosity, and vertical aortic annulus may not allow achieving CA; second, these techniques were only evaluated using a transfemoral approach; and finally, excessive catheter manipulation and rotation may increase the potential risk of embolic events and vascular complications.

## Imaging for THV commissural alignment assessment

### Computed tomography

CT is the gold standard for CA evaluation with direct measurement of the angle between the THV commissural post and native aortic valve commissure. The commissural offset is directly measured using an angle tool with the limbs aligned with the native commissure between the NCC and LCC and with the closest THV commissure. CA has been defined by the ALIGN-TAVR (Alignment of Transcatheter Aortic-Valve Neo-Commissures) consortium according to the commissural offset using a four-tier scale, as follows: aligned (0°–15°), mildly misaligned (15°–30°), moderately misaligned (30°–45°), and severely misaligned (45°–60°) (Figure 1) (6). However, although CT is the gold standard, this requires additional contrast injection and irradiation and may only be used as a postprocedural evaluation tool.

### CT-based simulations

CT-based simulations have been shown to be feasible with the Acn2 THV to predict CA based on pre-TAVR CT and using a 3D printed model for *in vitro* simulations of patient-specific rotation of the delivery system on the basis of patient-specific anatomy (14). CT-based simulations may also allow defining a crimping position for balloon-expandable THVs to achieve CA (12). CT-based simulations allow us to plan for the procedure and predict CA prior the TAVR procedure. However, CT-based simulations require dedicated software and may not be routinely used.

### Fluoroscopy

Several fluoroscopic methods have been described to assess for CA. Fluoroscopic methods may help to optimize for CA during the TAVR procedure and to assess for CA immediately following the procedure, avoiding post-TAVR CT.

One of the first described fluoroscopic methods was based on a CT-fluoroscopic overlay (15). Although this method demonstrated a good correlation with post-TAVR CT, this required identical patient positioning on the CT table during the angiographic procedure and CT/fluoroscopy coregistration, which might be challenging in some cases.

More recently, methods based on CT-derived fluoroscopic views have been proposed using both the 3-cusp view and cusp overlap views to align one of the THV commissural posts with the isolated native commissure (Figure 1) (11). Ultimately, although these methods are reproducible, they only provide a qualitative assessment of CA. Quantitative assessment of CA with S3 has been demonstrated by Akodad et al*.* using trigonometry to calculate the commissural offset between the posterior commissure (between the LCC and NCC) and the closest THV commissure (commissural offset angle θ = arcsine projected distance of commissural strut to centerline/projected radius). This method was shown to have an excellent correlation with post-TAVR CT (16). Although promising, all these fluoroscopic methods require a standardized methodology for reproducibility.

## Clinical impact of commissural misalignment

### Coronary access

In addition to tall frame THV design with supra-annular leaflets and high implants, severe commissural misalignment is a well-known independent predictor of unsuccessful selective coronary access following TAVR with a supra-annular THV (6, 17). Although coronary access might be less challenging in short-frame THVs, post-TAVR CT studies have demonstrated that around one-third of S3 THV stent frames extend beyond the coronary ostia (7). The RE-ACCESS (Reobtain Coronary Ostia Cannulation Beyond Transcatheter Aortic Valve Stent) prospective study found an overall 7.7% rate of unsuccessful selective coronary cannulation, almost exclusively following implantation of Evolut R or Evolut Pro supra-annular THVs. In addition, high THV implant and THV oversizing were associated with unsuccessful cannulation (18). However, as demonstrated in the ALIGN-ACCESS (TAVR with Commissural Alignment Followed by Coronary Access) study, the CA of supra-annular THVs, which can be achieved with the Evolut platforms, improves the feasibility of selective coronary access (7). Challenging coronary access might be even more frequent in valve-in-valve and redo-TAVR procedures, especially in the case of commissural misalignment and initial high implant of the initial THV, increasing the neo-skirt height (6, 19, 20).

### Repeatability

Redo-TAVR might be an increasingly performed procedure as the age at the first TAVR procedure tends to decrease in a lower-risk population with longer life expectancy. The risks of coronary obstruction and challenging coronary access following redo-TAVR are well documented. Indeed, a recent CT simulation study has demonstrated that around one-third of patients have a high risk of coronary obstruction following TAVR, regardless of the THV type, with this risk being increased if the failed THV is a self-expandable platform (4). The chimney technique is a well-known bailout strategy to prevent or treat coronary obstruction (21). Leaflet modifications techniques including bioprosthetic or native aortic scallop intentional laceration to prevent iatrogenic coronary artery obstruction (BASILICA) may also decrease the risk of acute coronary obstruction and facilitate future coronary access in patients undergoing valve-in-valve or redo-TAVR identified at high risk for coronary occlusion (22). However, these techniques are only feasible in the case of acceptable CA and may be irrelevant in the case of overlap between the THV or bioprosthetic valve commissural posts and the coronary ostia. Moreover, if the coronary ostia are located too close to the failed THV commissural post, this may lead to unsuccessful BASILICA with coronary obstruction (6).

### THV hemodynamics

Although future coronary access is the main concern when considering commissural misalignment, other concerns have been raised, especially the impact of commissural misalignment on THV function, durability, and risk of HALT (6). Indeed, significant commissural misalignment may lead to non-physiological flow with increased leaflet stress and blood stagnation inside the sinuses of Valsalva and eventually increased thrombogenicity and early THV deterioration (6, 23). A bench study assessing the impact of commissural misalignment on THV hydrodynamics using the Sapien XT device failed to demonstrate any impact of commissural misalignment on THV hydrodynamics but showed impaired fluid flow patterns in the case of THV misalignment (24). Another bench study with the Acurate neo THV in a valve-in-valve configuration demonstrated that THV commissural misalignment was associated with a higher incidence of paravalvular leakage and impaired leaflet motion due to interaction with the leaflets of the surgical valve (25). Recently, an association between severe commissural misalignment and an early increase in gradient following an S3 THV implant have been suggested (26). Data from the RESOLVE registry among 324 patients showed that significant commissural misalignment defined as commissural offset > 30° was observed in 52.8% of patients but was not associated with significant paravalvular leakage, intravalvular leakage, increase in the mean gradient >20 mmHg, or HALT initially. However, significant commissural misalignment was associated with twice as much significant increase in THV mean gradient (>50% relative increase) from discharge to 1 month (17.6% vs. 8.26%; *p* = 0.038) compared with commissural alignment <30° and with a linear relationship (1.3-fold risk of a significant increase in mean gradient every 10° of commissural misalignment) (26). A significantly higher rate of mild valvular regurgitation has been recently described in patients with at least moderate commissural misalignment (27). The incidence of HALT was higher with the S3 THV in the case of commissural misalignment (40% vs. 28% in case of CA) in a recent report (28). Although unfavorable THV hemodynamics and blood stagnation have been advocated as potentially increasing the risk of reduced THV durability, no clear evidence exists, and further investigations are required.

## Discussion

Commissural alignment is becoming one of the cornerstones in TAVR when considering the lifetime management of aortic stenosis in younger populations. Progress was made aligning commissures with most of the available THV platforms to date, and assessment of CA using CT and fluoroscopy has now shown to be accurate. However, although CA represents one step in the right direction for TAVR procedure optimization, a certain number of limits remain (Figure 1).

## Limitations

### Coronary eccentricity

Although CA appears to be a reasonable goal to achieve, it might not always reflect alignment between the THV and coronary ostium. Indeed, coronary eccentricity with the coronary ostium not being centered in the corresponding cusp has been described recently and is more frequent with the right coronary artery than the left main, which may lead to coronary overlap despite adequate commissural alignment (29, 30). New approaches have been proposed using CT-derived (coronary ostial overlap) views to better understand the relationship between the THV commissures and coronary ostia (30) with promising results. Moreover, leaflet modification techniques may be more efficient in the case of coronary alignment than commissural alignment (29, 30).

### Bicuspid aortic valve

A recent CT study has shown that cusp asymmetry was more frequent in patients with type 1 bicuspid aortic valves (69%) compared with tricuspid aortic valves (17.5%), the dominant cusp being the NCC in the majority of bicuspid cases (30). Thus, CA might be more challenging with bicuspid aortic valves, especially in the case of left-right cusps fusion. Depending on the type of bicuspid valve, CA might be achieved using the same techniques as for tricuspid valves, or advanced catheter techniques may be used based on pre-TAVR CT analysis. Coronary alignment rather than CA might be more accurate, especially in bicuspid anatomies. Patient-specific simulation may be useful to avoid overlap between THV commissures and coronary ostia.

### Future perspectives

CT-based patient-specific simulation, although not routinely performed, may help to better understand and plan for specific device orientation or crimping position to achieve optimal CA (13, 14). This represents a useful tool, especially for lifetime management in younger patients. Along with TAVR procedure improvement, new TAVR devices with markers aligned with the THV commissures and improved torquability and self-rotation may allow a more accurate commissural alignment prior to deployment, especially for balloon-expandable THVs. Future investigations are still required to assess the impact of commissural misalignment on long-term outcomes following TAVR.

## Conclusion

Commissural alignment is becoming an important parameter for procedure optimization in younger patients undergoing TAVR. Although progress has been made to improve CA with the current device and CA understanding during the TAVR procedure, improvement in implant techniques and devices is expected.

## Author contributions

MA: conceptualization, data curation, investigation, visualization, and writing—original draft. YL: data curation, data curation. DM: data curation, review. FS: data curation, review. TH: data curation, review. PB: data curation, review. JS: data curation, review. GT: data curation, review. JL: data curation, review. DW: data curation, review. JW: review. BC: validation, review, and editing. All authors contributed to the article and approved the submitted version.

## Conflict of interest

MA has received research funding from Medtronic, Biotronik, MUSE Explore, and Federation Française de Cardiologie. DM is supported by the Swiss National Science Foundation (grant no. P2LAP3_199561) and SICPA. PB is a consultant to Edwards Lifesciences and provides CT core lab services to Edwards Lifesciences, Medtronic, Neovasc, Boston, and Abbott, for which no direct compensation is received. JL is supported by a Canadian Research Chair in Advanced Cardiopulmonary Imaging, a consultant for MVRX, Heartflow Inc., and Circle Cardiovascular Imaging, and provides CT core lab services to Edwards Lifesciences, Medtronic, Neovasc, Boston Scientific, and Tendyne Holdings, for which no direct compensation is received. JS has received speaking fees from Edwards Lifesciences and is a consultant to Edwards Lifesciences, Boston Scientific, NVT Medical, and Medtronic. GT is supported by the Fondation Vaudoise de Cardiologie and the SICPA Foundation. DW is a consultant to, and has received research funding from, Edwards Lifesciences and Abbott. JW is a consultant to Edwards Lifesciences and Abbott. BC is a minor shareholder of Colibri and CERC. The remaining authors declare that the research was conducted in the absence of any commercial or financial relationships that could be construed as a potential conflict of interest.
